# Audit on Availability, Quality and Frequency of Clinical Supervision

**DOI:** 10.1192/bjo.2022.425

**Published:** 2022-06-20

**Authors:** Elizabeth Askew, Gayathri Gnanasekaram, Amanda Hoar

**Affiliations:** Somerset NHS Foundation Trust, Taunton, United Kingdom

## Abstract

**Aims:**

We have completed a cycle of audit on the availability and quality of clinical supervision in Somerset NHS Foundation Trust. Last year we had highlighted the results of our first survey (run in 2020) in local teaching and audit meetings. We have now completed the cycle following the intervention. Both Severn deanery and Somerset NHS Foundation Trust both recommend psychiatry trainees have one hour of supervision per week, involving exploration of trainee clinical and educational needs. This audit is now part of a quality improvement project being run across Severn Deanery. This particular audit focuses on the results from Somerset NHS Foundation Trust.

**Methods:**

Trainees working in Somerset NHS Foundation Trust were invited to participate in this survey. We used the original survey from last year but added further white spaces to invite feedback and to explore what was particularly good about the clinical supervision currently offered. Questions on accomplishing workplace based assessments (WPBA), managing e-portfolio requirements were asked, with Likert scale responses available. The survey was sent out in the form of Microsoft Forms disseminated via email to all junior doctors (n = 27).

Survey was run from May till June 2021 (nearing the end of placement). We sent out 3 reminders before closing the survey. The authors of the audit then reviewed the data.

**Results:**

9 out of 27 doctors responded, response rate of 33%. Our last survey had a response rate of 63%. Supervision appears to be more regular now with only 11% stating that they were meeting their supervisor sometimes in comparison to 17% the last survey. Similar percentage of respondents were able to complete WBPAs as in the last survey (88% Vs 89%).

QI project/audits were being discussed at a similar rate (60% Vs 66%). 75% of psychiatry trainee respondents were discussing their psychotherapy competencies (42% were having some discussion in the last survey). There was a better response from GP and FY doctors for this survey.

**Conclusion:**

Response rate appears to have fallen, however supervision appears to be more regular with more focus on competencies.

White space answers showed that most trainees were satisfied with supervision. However, supervision could be more consistent and serious attempts must be made to protect it from clinical work overshadowing it.

We will be comparing the results of our audit in Somerset NHS Foundation Trust to the results from other parts of the deanery.

